# The burden of gynecomastia among men on antiretroviral therapy in Zomba, Malawi

**DOI:** 10.1371/journal.pone.0188379

**Published:** 2017-11-20

**Authors:** Victor Singano, Alemayehu Amberbir, Daniela Garone, Christopher Kandionamaso, Jack Msonko, Monique van Lettow, Kondwani Kalima, Yamikani Mataka, Gift Kawalazira, Gabriel Mateyu, Aunex Kwekwesa, Alfred Matengeni, Joep J. van Oosterhout

**Affiliations:** 1 Dignitas International, Zomba, Malawi; 2 Dala Lana School of Public Health, University of Toronto, Toronto, Canada; 3 Zomba District Health Office, Malawi Ministry of Health, Zomba, Malawi; 4 Department of Medicine, College of Medicine, Blantyre, Malawi; University of Cape Town, SOUTH AFRICA

## Abstract

**Background:**

Many Africans who are on life-saving ART face challenges from a variety of toxicities. After the introduction of a standardized first-line efavirenz-containing ART regimen, reports of gynecomastia appeared in Malawian popular media, however data on the prevalence and risk factors of gynecomastia from Africa are lacking.

**Methods:**

We conducted a cross–sectional study in males ≥18 years registered on ART at the HIV clinic in Zomba Central Hospital. Men who reported to have ever experienced breast or nipple enlargement received a standard questionnaire and underwent physical examination. Questions included perceptions and concerns about gynecomastia. Clinicians confirmed the presence and severity of gynecomastia. Routinely collected data on current and previous ART regimens, CD4 count, WHO clinical stage, anthropometric measurements and history of tuberculosis were extracted from the electronic database.

**Results:**

We enrolled 1,027 men with median age 44 years (IQR: 38–52). The median ART duration was 57 months (IQR: 27–85); 46.7% were in WHO stage III/IV at ART initiation, 88.2% had exposure to efavirenz and 9% were overweight or obese. The prevalence of self-reported gynecomastia was 6.0% (62/1027) (95%-CI: 4.7–7.7%). Of men with gynecomastia 83.6% reported nipple enlargement and 98.4% enlarged breasts (85.5% bilateral). One-third said they had not reported gynecomastia to a health care worker. Over three-quarters mentioned that gynecomastia was an important or very important problem for them, while more than half were embarrassed by it. On examination gynecomastia was present in 90% (confirmed gynecomastia prevalence 5.5%; 95%-CI: 4.2–7.0%) and 51.8% had severity grade III or IV. History of tuberculosis treatment was independently associated with self-reported gynecomastia, adjusted OR 2.10 (95%-CI: 1.04–4.25).

**Conclusions:**

The burden of gynecomastia among men on ART in Malawi was higher than previously reported, and was associated with adverse psychological consequences, calling for increased awareness, a proactive diagnostic approach and diligent clinical management.

## Introduction

The prognosis of HIV infection in sub-Saharan Africa has improved dramatically since the introduction of antiretroviral therapy (ART). Adverse effects of ART remain a major challenge for adherence and treatment success [1–4]. Gynecomastia is defined as breast enlargement due to benign proliferation of glandular tissue [5] and has been recognized as a side effect of efavirenz [6,7], constituent drug of most current standardized first-line ART regimens in the region [8].

The literature on HIV-associated gynecomastia mainly consists of case reports and -series. In few larger surveys from high-income countries it has been estimated that the prevalence of gynecomastia in HIV infected males is around 3% [9–11]. There are very few reports from Africa regarding HIV-associated gynecomastia and the prevalence of gynecomastia in HIV infected Africans is unknown. In Malawi, routine side effect monitoring of ART in the national HIV programme strongly underreports the true prevalence of toxicities, because health care workers generally only report a toxicity at the time that a change of ART regimen is required [12].

After the introduction of a new standardized first-line ART regimen in Malawi consisting of tenofovir/lamivudine/efavirenz in 2011–2012, there were several news articles and reports in popular media about breast enlargement in males which created some controversy and negative publicity about ART [13]. In response, we reported case studies of two patients with gynecomastia from Zomba district, Malawi with recommendations for clinical management in the local setting [13]. In a rapid survey in 18 health facilities in Mangochi district, Malawi we identified 114 cases of gynecomastia among men on ART. Because a denominator could not be determined formally, this survey did not allow determination of a reliable prevalence estimate [14], information that is important for HIV management policy in national and regional programmes.

We therefore conducted a cross-sectional study among male adults on ART in Zomba, Malawi to determine the prevalence of gynecomastia and explore factors associated with it.

## Methods

### Study setting and participants

This cross-sectional study was conducted at the urban Tisungane HIV clinic of Zomba Central Hospital in Zomba district, south-east Malawi where adult HIV prevalence is around 15% [15]. The clinic had an estimated 6,500 patients on ART at the time of the study, including around 2,200 men. The study was conducted from March through June 2016. All male patients were invited to participate in the study on arrival at the clinic by reception staff. During the study period all men attending the ART clinic were attended to by study clinicians in a single dedicated consultation room. Male patients were invited into the study if aged ≥18 years and registered for ART services at Zomba Central Hospital, regardless of duration of treatment. We excluded patients who had been referred from other clinics. The study was approved by the College of Medicine Research Ethics Committee (protocol number P.11/14/1686) and written informed consent was obtained from all study participants. Patients with gynecomastia were managed according to Malawi HIV guidelines.

#### Data collection

Study clinicians followed a standardized protocol. Participants were asked if they had ever experienced breast or nipple enlargement. Patients who replied “no” continued the routine HIV clinic consultation while to those who answered “yes” we administered a questionnaire to collect further details about associated symptoms, co-medications and perceptions on gynecomastia. Although not formally validated, the questionnaire was adapted after piloting it at the HIV clinic. This was followed by a physical examination to confirm self-reported gynecomastia, and determine severity grading according to American Society of Plastic Surgeons. Physical examination was not blinded to the self-report of gynecomastia. The most advanced stages in this classification are defined as follows: moderate breast enlargement that exceeds areola boundaries, with edges that are distinct from the chest with skin redundancy (stage III) and marked breast enlargement with skin redundancy and feminization of the breasts (stage IV) [16]. Clinicians also looked for the presence of lipodystrophy [17], signs of liver disease (ascites, jaundice, hepatomegaly) and testicular masses. Data on current and previous ART regimens, age, weight, height, history of tuberculosis treatment, baseline CD4 count and WHO clinical stage and duration of ART were extracted from the electronic medical record system of the clinic for all participants, whether with self-reported gynecomastia or not. History of TB treatment only refers to information about TB treatment that was started at or within one year before the initiation of ART. Information about incident TB during ART was not available to us.

### Sample size and precision

We planned enrolling consecutive male patients throughout the predefined study period of 4 months. With a presumed gynecomastia prevalence of 3%, a sample size of 1,000 provides a precision of 0.01, meaning a 95% confidence interval (CI) of plus or minus 1% around the prevalence.

### Data management and analysis

Data were verified daily for completeness and entered in an Access database, cleaned, coded, compared against the original case report form, and merged for analysis using Stata 13 (Statacorp, College Station Texas, USA). We used descriptive statistics to present patients’ demographic and clinical characteristics and their perceptions of gynecomastia. Multivariable logistic regression analyses were performed to determine the independent associations of demographic and clinical characteristics with confirmed gynecomastia. A secondary analysis was done with all the men who reported gynecomastia, including those without abnormalities on examination. Plausible multi-collinearity was assessed and variables with a p-value of ≤0.1 in univariate analyses were included in the multivariable logistic regression models. Participant’s age, exposure to efavirenz, exposure to stavudine and history of TB treatment were also included in the model as a priori confounders, irrespective of p-values in the univariable analysis, given their biological importance in relation to gynecomastia in previous literature. The significance of the association between patients’ characteristics and gynecomastia was assessed using the Wald statistic to determine p-values. To determine if there was an age trend in relation to the outcome, p-value for trend (p-trend) was computed for participant’s age in the model. We also reported an overall p-value for age categories (18–34, 35–44, ≥44 years) using a likelihood ratio test.

## Results

### Study enrolment

A total of 1,027 participants were included in the study (Table 1), which was 48% of all adult men on ART in care at the time of the study. The reason why half the target population did not enroll was not recorded but several are possible: patients can send registered relatives to collect medication on their behalf, men attended other clinics for ART services (a research clinic, the integrated TB-ART clinic, the health care workers clinic), the team may have missed men who attended the clinic, men may have avoided screening and enrolment due to worries about additional waiting time, and there may be a general reluctance among men to enroll into studies. However, none of the men who were invited to participate withheld consent. Because we noticed that consecutive enrolment of all attending male patients did not happen as planned, we determined whether the study population was representative of the overall male clinic population aged ≥18 years by comparing routinely collected patient characteristics of participants and non-participants. As described in S1 Table, we found significant differences: participants were older, had lower body mass index (BMI), had received tuberculosis treatment less frequently, and were more commonly (7.2% vs. 3.5%) on the ART regimen tenofovir/lamivudine/nevirapine, which is the regimen that is recommended after efavirenz toxicity.

**Table 1:** Demographic and clinical characteristics of men on ART screened for gynecomastia at Zomba Central Hospital (N = 1027).

Characteristic	Number (%)
*Total number of participants enrolled*	1027
*Age*, *years (%) (N = 1027)*	
18–34	130 (12.7)
35–44	389 (37.9)
≥44	508 (49.5)
*Median age*, *years (IQR) (N = 1027)*	44 (38–52)
*Body Mass Index*, *Kg/m*^*2*^ *(%) (N = 1027)*	
Underweight	156 (15.2)
Normal	779 (75.9)
Overweight	79 (7.7)
Obese	13 (1.3)
*Median Body Mass Index*, *Kg/m*^*2*^ *(IQR) (N = 1027)*	20.8 (19.3–22.5)
*CD4 count at ART initiation*, *cells/mm*^*3*^ *(%) (N = 718)*	
CD4 < 250	446 (62.1)
CD4 ≥250	272 (37.9)
*Median CD4 count at ART initiation (IQR) (N = 718)*	199 (99–312)
*WHO disease stage at ART initiation (%) (N = 812)*	
Stage I	211 (26.0)
Stage II	222 (27.3)
Stage III	296 (36.5)
Stage IV	83 (10.2)
*History of TB treatment (%) (N = 1027)*	
Yes	91 (8.9)
No	936 (91.1)
*Presence of lipodystrophy (%) (N = 1027)*	
Yes	70 (6.8)
No	957 (93.2)
*Current ART regimen (%) (N = 1026)*	
zidovudine/lamivudine/nevirapine	90 (8.8)
tenofovir/lamivudine/efavirenz	826 (80.5)
tenofovir/lamivudine + nevirapine	74 (7.2)
Other regimens*	36 (3.5)
*ART duration (%) (N = 1027)*	
<24 months	229 (22.3)
≥ 24 months	789 (77.7)
*Median ART duration*, *months (IQR)*	57 (27–85)
*Number of ART regimens (%) (N = 1027)*	
One	328 (31.9)
Two	556 (54.1)
Three	123 (12.0)
Four or more	20 (2.0)
*Exposure to efavirenz (%) (N = 1026)*	
No	121 (11.8)
Yes	905 (88.2)
*Exposure to stavudine (%) (N = 1026)*	
No	357 (34.8)
Yes	669 (65.2)

SD, standard deviation; IQR, interquartile range; ART, antiretroviral therapy

*Other regimens: stavudine, lamivudine, nevirapine; tenofovir, lamivudine plus atazanavir/ritonavir; zidovudine, lamivudine plus atazanavir/ritonavir

### Demographic and clinical characteristics of adult men on ART

The median age was 44 years (inter-quartile range [IQR]: 38–52). At the start of ART, the median CD4 count was 199 cells/mm^3^ (IQR: 99–312) and 53.3% were in the WHO clinical stages I or II. At study enrolment, 9.0% participants were overweight or obese, 8.9% had ever been treated for tuberculosis, and 6.8% had signs of lipodystrophy. The median ART duration was 56.9 months (IQR: 27–85); the prevalence of exposure to stavudine was 65.2% and to efavirenz 88.2% (Table 1).

### Characteristics of men on ART with gynecomastia

There were 62 cases of self-reported gynecomastia, resulting in a prevalence of 6.0% (95% CI: 4.7–7.7%) of whom 51 (84.6%) reported nipple enlargement and 61 (98.4%) breast enlargement. Of the self-reported cases, 66.7% said they had ever reported symptoms to a health care worker. Painful breasts was a common symptom (41.9%), nipple discharge had ever occurred in 6.5%. Prevalence of exposure to stavudine was 74.3% and to efavirenz 90.2%. In 56 cases (90.3%), gynecomastia was present on physical examination, therefore the prevalence of confirmed gynecomastia was 5.5% (95%-CI: 4.2–7.0%). No cases of lipomastia were observed. The six men who reported gynecomastia that was not confirmed on examination had substituted efavirenz for nevirapine at least one year before the study, with documentation in the routine monitoring tools as side effect “other” (n = 4) or without documentation of the reason for changing therapy (n = 2). At the time of the study gynecomastia could not be reported specifically in routine ART monitoring tools. It is therefore likely that gynecomastia had resolved after substitution of efavirenz in these six patients. Gynecomastia severity grade III or IV was present in 51.8%. Twelve patients (19.4%) had signs of lipodystrophy; none had signs of liver disease and no testicular masses were found on examination (Table 2).

**Table 2:** Characteristics of gynecomastia cases among men on ART at Zomba Central Hospital.

Patient characteristics	Number (%)
*Total number of gynecomastia cases*	62 (6.0)
*Self-report of nipple enlargement (n = 61)*	
No nipple enlargement	10 (16.4)
Right nipple	3 (4.9)
Left nipple	4 (6.6)
Both nipples	44 (72.1)
*Self-reported of breast enlargement (n = 62)*	
No breast enlargement	1 (1.6)
Right breast	4 (6.5)
Left breast	4 (6.5)
Both breasts	53 (85.5)
*Ever reported breast enlargement to clinic staff (n = 57)*	
Yes	38 (66.7)
No	19 (33.3)
*Gynecomastia grading (n = 56)*	
Grade 1	9 (16.1)
Grade 2	18 (32.1)
Grade 3	9 (16.1)
Grade 4	20 (35.7)
*Pain associated with gynecomastia (n = 62)*	
Yes	26 (41.9)
No	36 (58.1)
*Nipple discharge associated with gynecomastia (n = 62)*	
Yes	4 (6.5)
No	58 (93.6)
*History of TB treatment (n = 62)*	
Yes	10 (16.4)
No	51 (83.6)
*Presence of lipodystrophy (n = 62)*	
Yes	12 (19.4)
No	50 (80.7)
Signs of liver disease* *(n = 62)*	
Yes	0 (0)
No	62 (100)
*Testicular mass on examination (n = 61)*	
Yes	0 (0)
No	61 (100)
*Co-medications (n = 36)*	
Isoniazid	1 (1.6)
Cotrimoxazole	35 (56.5)
Missing	26 (41.9)
*Current ART regimen (n = 62)*	
stavudine/lamivudine/nevirapine	1 (1.6)
tenofovir/lamivudine/efavirenz	9 (14.5)
tenofovir/lamivudine + nevirapine	48 (77.4)
zidovudine/lamivudine + atazanavir/ritonavir	4 (6.5)
*Exposure to efavirenz (n = 62)*	
No	6 (9.7)
Yes	56 (90.3)
*Exposure to stavudine (n = 62)*	
No	16 (25.8)
Yes	46 (74.2)
*Number of ART regimens (n = 62)*	
One	6 (9.7)
Two	10 (16.1)
Three	38 (61.3)
Four or more	8 (12.9)

*Presence of liver disease assessed using signs of ascites, jaundice and hepatomegaly

### Patients’ perceptions of gynecomastia

Forty-eight patients (77.5%) said that gynecomastia was an important or very important problem for them. Embarrassment was reported by 32 (51.6%), stigma by 6 (9.7%) and 44 (71.0%) were concerned that gynecomastia affected their health. Only 4 (6.6%) patients reported skipping ART doses because of gynecomastia and 45 (72.6%) patients felt that gynecomastia was the result of an ART side effect (Table 3).

**Table 3:** Perceptions of gynecomastia among affected men on ART in Zomba Central Hospital.

Perception question	Number (%)
*How important a problem is gynecomastia for you*? *(n = 62)*	
Very important	28 (45.2)
Important	20 (32.3)
Less important	10 (16.1)
Not important	4 (6.5)
*Are you concerned that your breast symptom affects your health*? *(n = 62)*	
Yes	44 (71.0)
No	18 (29.0)
*Are you embarrassed by your gynecomastia*? *(n = 62)*	
Yes	32 (51.6)
No	30 (48.4)
*Do you feel stigmatized due to your gynecomastia*? *(n = 62)*	
Yes	6 (9.7)
No	56 (90.3)
*According to you*, *is gynecomastia a side effect of ART*? *(n = 62)*	
Yes	45 (72.6)
No	17 (27.4)
*Have you skipped ART pills due to your gynecomastia*? *(n = 59)*	
Yes	4 (6.6)
No	55 (90.1)

### Factors associated with gynecomastia

In univariate analyses, no significant associations were observed between confirmed gynecomastia and age, body mass index, CD4 count and WHO disease stage at ART initiation, history of tuberculosis, duration on ART, and presence of lipodystrophy. The current ART regimen and the number of ART regimens since the start of treatment were associated with gynecomastia, but because both variables are directly related to a change of regimen upon diagnosis of gynecomastia, they were not included in multivariable models and no factors were independently associated with confirmed gynecomastia in the multivariable analysis (Table 4). In a secondary analysis we also included men with self-reported gynecomastia which was not confirmed on physical examination, i.e. in whom gynecomastia had probably resolved after substitution of efavirenz to nevirapine. In this analysis, history of tuberculosis was associated with gynecomastia in uni- and multivariable analyses, adjusted Odds Ratio (aOR) 2.10 (95% CI: 1.04–4.25; p = 0.04) (S2 Table).

**Table 4:** Factors associated with confirmed gynecomastia among men on ART at Zomba Central Hospital.

Characteristics	Gynecomastia					
	Total N (%)	n (%) Gynecomastia	Crude OR (95% CI)	P—value	Adjusted OR (95% CI)*	P—value^¥^
*Age in years (N = 1027)*				0.354		0.556 P_trend_ 0.338^§^
18–34	130 (12.7)	4 (3.1)	1		1	
35–44	389 (37.9)	21 (5.4)	1.80 (0.61, 5.34)	0.291	1.56 (0.52, 4.69)	0.429
≥44	508 (49.5)	31 (6.1)	2.05 (0.71, 5.91)	0.185	1.75 (0.59, 5.15)	0.311
*BMI categories (N = 1025)*				0.732		
Underweight	155 (15.1)	10 (6.5)	1.27 (0.62, 2.60)	0.509		
Normal	778 (75.9)	40 (5.1)	1			
Overweight	92 (9.0)	6 (6.5)	1.29 (0.53, 3.12)	0.577		
*Baseline CD4 count at ART initiation (N = 718)*				0.162		0.292
CD4 < 250	446 (43.4)	28 (6.3)	1		1	
CD4 ≥250	272 (26.5)	9 (3.3)	0.51 (0.24, 1.10)	0.086	0.58 (0.26, 1.29)	0.179
Missing	309 (30.1)	19 (6.2)	0.98 (0.54, 1.78)	0.942	1.06 (0.57, 1.94)	0.862
*ART duration in months (N = 1027)*						
<24	229 (22.3)	9 (3.9)	1			
≥24	798 (77.7)	47 (5.9)	1.53 (0.74, 3.17)	0.253		
*WHO stage at ART initiation (N = 812)*				0.526		
Stage I/II	433 (42.2)	23 (5.3)	1			
Stage III/IV	379 (36.9)	24 (6.3)	1.21 (0.67, 2.17)	0.535		
Missing	215 (20.9)	9 (4.2)	0.78 (0.35, 1.71)	0.534		
*History of TB treatment (N = 1027)*						0.258
No	936 (91.1)	48 (5.1)	1		1	
Yes	91 (8.7)	8 (8.8)	1.78 (0.82, 3.90)	0.147	1.58 (0.71, 3.51)	
*Presence of lipodystrophy (N = 1027)*						
No	957 (93.2)	51 (5.3)	1			
Yes	70 (6.8)	5 (7.1)	1.37 (0.53, 3.54)	0.536		
*Current ART regimen (N = 1026)*				**<0.001**		
zidovudine/lamivudine/nevirapine	90 (8.8)	0 (0.0)	**-**			
tenofovir/lamivudine/efavirenz	826 (80.5)	9 (1.1)	**1**			
tenofovir/lamivudine + nevirapine	74 (7.2)	42 (56.8)	**119.15 (53.43, 265.67)**	**<0.001**		
other regimens**	36 (3.5)	5 (13.9)	**14.64 (4.63, 46.27)**	**<0.001**		
*Patient’s exposure to efavirenz (1*,*026)*						0.324
No	121 (11.8)	5 (4.1)	1		1	
Yes	905 (88.2)	51 (5.6)	1.38 (0.54, 3.54)	0.496	1.61 (0.62, 4.17)	
*Patient’s exposure to stavudine (n = 1*,*026)*						0.601
No	357 (34.8)	15 (4.2)	1	0.198	1	
Yes	669 (65.2)	41 (6.1)	1.49 (0.81, 2.73)		1.19 (0.62, 2.25)	
*Number of ART regimens (N = 1027)*				**<0.001**		
One	328 (31.9)	6 (1.8)	**1**			
Two	556 (54.1)	9 (1.6)	**0.88 (0.31, 2.50)**	0.815		
Three	123 (12.0)	34 (27.6)	**20.50 (8.34, 50.38)**	**<0.001**		
Four or more	20 (2.0)	7 (35.0)	**28.90 (8.50, 98.20)**	**<0.001**		

*Final model included: age, baseline CD4 count, history of TB treatment, patient’s exposure to efavirenz and patient’s exposure to stavudine

**Other regimens: stavudine, lamivudine, nevirapine; tenofovir, lamivudine, atazanavir/ritonavir and zidovudine, lamivudine, atazanavir/ritonavir

^¥^p-values are based on the Wald test, except the p-value for the association with age categories, which is based on the likelihood ratio test;

^§^ P_trend_ = p-value for trend

## Discussion

In this cross-sectional study from a large ART cohort in south-east Malawi we found that the prevalence of self-reported and confirmed gynecomastia among men on ART (6.0 and 5.5% respectively) was higher than previously reported. Gynecomastia was associated with perceived adverse consequences, including health concerns and embarrassment. A history of tuberculosis treatment resulted in a 2.1-fold higher risk of self-reported gynecomastia.

Estimates of the prevalence of HIV associated gynecomastia are based on a small number of studies from western settings, finding prevalences of 1.8–3% [9,11,18]. To our knowledge there are no studies that have previously reported incidence or prevalence of gynecomastia in HIV infected Africans. A retrospective study from South Africa reported that gynecomastia was the fourth most common adverse drug reaction associated with ART between 2010 and 2014 [1]. Four and a half years after the introduction of a standardized first-line regimen that contains efavirenz, the prevalence of self-reported gynecomastia was 6.0% in our clinic. Six cases reported having had swollen breasts previously but had no abnormalities on inspection. The prevalence of confirmed gynecomastia at the time of the study was therefore slightly lower (5.5%). Patients with gynecomastia who did not report this to the study staff may have been missed, as we did not inspect patients without previous or current symptoms. Because our strategy to consecutively enroll all adult males visiting the clinic was not achieved, selection bias may have caused the study population not to be representative of the adult male population on ART. Given that study participants were twice as likely to be on an ART regimen that is recommended as the alternative first-line regimen following efavirenz toxicity, we may have overestimated the prevalence of gynecomastia.

While the strength of formal evidence for the association of gynecomastia with antiretroviral drugs is moderate [6,7], gynecomastia has been attributed to use of protease inhibitors [19–21], the nucleoside reverse transcriptase inhibitors stavudine and didanosine [6,7,22] and most commonly to the non-nucleoside reverse transcriptase inhibitor efavirenz [22–24]. Gynecomastia was not independently associated with efavirenz exposure in our study, possibly because nearly all participants were exposed. Alternatively, a lack of power may not have detected a weak association if it existed. In a Spanish study, gynecomastia first appeared between four to sixteen months on efavirenz-based regimens and regressed after a median of five months following efavirenz withdrawal [22]. In six Nigerian ART patients, gynecomastia developed after a mean of ten months on efavirenz. Complete resolution of gynecomastia within six to ten weeks after withdrawal of efavirenz was observed in five cases [25]. In six of our patients (9.7%) self-reported gynecomastia had apparently fully resolved and a large majority had persistent gynecomastia. However we did not collect data on the time to resolution or on the duration of persistence after a change to nevirapine-based ART. Gynecomastia has been linked to other drugs that are in regular use in our setting such as isoniazid [26], spironolactone and ketoconazole [27]. We found that a history of tuberculosis treatment containing isoniazid was independently associated with gynecomastia, but only in the secondary analysis which included the six cases without objective gynecomastia at the time of the study. Our ability to get adequate information about long-term usage of relevant other drugs was very limited and we cannot draw conclusions concerning associations with other than anti-tuberculous and antiretroviral drugs. The higher prevalence of gynecomastia that we report in comparison with western studies may be due to more frequent exposure to isoniazid containing tuberculosis treatment.

Gynecomastia has been associated with chronic liver disease and testicular tumors [5,27] but we found no testicular masses or signs of liver disease among patients with gynecomastia. The sensitivity of physical examination to rule out testicular tumors and especially chronic liver disease may not be high, but our findings question the relevance of routine physical examination for this purpose in our setting. We found that 19.4% of gynecomastia cases had signs of lipodystrophy, a combination of toxicities that has been observed regularly. Efavirenz induced gynecomastia was described in six French patients with lipodystrophy syndrome [24] and in a Spanish study lipoatrophy was independently associated with gynecomastia [18]. It is important to distinguish between gynecomastia and lipomastia, which is breast swelling due to fat accumulation as seen in lipodystrophy [28,29], through detailed history and breast examination [5], because the management of both conditions is different. Breast pain was a commonly associated symptom (41.9%) in our gynecomastia cases and was also reported in patients with HIV associated gynecomastia from Nigeria [25]. Individuals with nipple discharge, as reported by 6.5%, need further investigation because it may be a symptom of endocrinological disease or breast cancer [27].

The study findings must be interpreted in the light of several limitations, some have already been discussed. Perceptions about gynecomastia were based on a straight-forward, standardized questionnaire which may be prone to reporting bias. Lipodystrophy diagnosis was established by experienced clinicians with a validated tool, but used on a single occasion the determination may be less reliable. We were unable to diagnose hypogonadism which is an important cause of gynecomastia in HIV infected men. We did not collect data about the exact time that gynecomastia developed in relation to various drug exposures, nor could we observe clinical progression and improvement. The cross sectional design of our study limits conclusions about the causality of the observed associations. Finally, this was a single-center study in a tertiary urban clinic and results may not be extrapolated to other settings.

Our results have important consequences for the approach to gynecomastia in clinical practice. Clinicians should proactively ask about gynecomastia during clinic visits given that it is a regularly occurring side effect in men on the current first-line ART regimen and up to one-third did not report to health care workers that they had gynecomastia, possibly related to embarrassment. Misconceptions and incomplete knowledge among patients need to be addressed through counseling sessions before the start of ART and during follow up. Because gynecomastia was an important health concern for most patients and a relevant percentage was embarrassed, stigmatized or skipped ART doses, active management is needed, including timely switching to alternatives for efavirenz.

## Conclusions

In the era of efavirenz containing standardized first-line ART, the burden of clinician-confirmed and self-reported gynecomastia among adult male ART patients in Malawi was 5.5% and 6.0% respectively, higher than in previous studies from western settings. Tuberculosis treatment was the only factor that was independently associated with self-reported gynecomastia. Gynecomastia was associated with adverse individual consequences, calling for increased awareness, a proactive diagnostic approach and diligent clinical management.

## Supporting information

S1 Table Men on ART at Zomba Central Hospital (N = 2,145): Comparing demographic and clinical characteristics of study participants and non-participants. (DOCX)

S2 Table (DOCX)
