# Supplementary material for: The burden of gynecomastia among men on antiretroviral therapy in Zomba, Malawi
Source: PLoS One. 2017 Nov 20;12(11):e0188379. doi: 10.1371/journal.pone.0188379 (PMC5695797; doi:10.1371/journal.pone.0188379)
Supplement: S1 Table — (DOCX) [file pone.0188379.s001.docx]

**Table S1.** Men on ART at Zomba Central Hospital (N=2,145): comparing demographic and clinical characteristics of study participants and non-participants

| **Characteristics** | **HIV cohort of men during the study period**  **N (%)** | **Non study participants**  **(N=1,118)**  **n (%)** | **Study participants (N=1,027)**  **n (%)** | **P-value** |
| --- | --- | --- | --- | --- |
| *Age in years (N=2,145)* |  |  |  | <0.001 |
| 18 – 34 | 380 (17.2) | 250 (22.4) | 130 (12.7) |  |
| 35 – 44 | 838 (39.1) | 449 (40.2) | 389 (37.9) |  |
| ≥44 | 927 (43.2) | 419 (37.5) | 508 (49.5) |  |
| *Body Mass Index, Kg/m^2^ (N=2,130)* |  |  |  | <0.001 |
| Underweight | 304 (14.3) | 149 (13.5) | 155 (15.1) |  |
| Normal | 1,561 (73.3) | 783 (70.9) | 778 (75.9) |  |
| Overweight | 221 (10.4) | 142 (12.9) | 79 (7.7) |  |
| Obese | 44 (2.1) | 31 (2.8) | 13 (1.3) |  |
| *WHO disease stage at ART initiation (N=1,926)* |  |  |  | 0.020 |
| Stage I | 553 (28.7) | 342 (30.7) | 211 (26.0) |  |
| Stage II | 474 (24.6) | 252 (22.6) | 222 (27.3) |  |
| Stage III | 681 (35.4) | 385 (34.6) | 296 (36.5) |  |
| Stage IV | 218 (11.3) | 135 (12.1) | 83 (10.2) |  |
| *History of TB treatment (N=2,145)* |  |  |  | 0.002 |
| Yes | 237 (11.1) | 146 (13.1) | 91 (8.9) |  |
| No | 1,908 (89.0) | 972 (86.9) | 936 (91.1) |  |
| *Presence of lipodystrophy (N=2,145)* |  |  |  | 0.585 |
| Yes | 153 (7.1) | 83 (7.4) | 70 (6.8) |  |
| No | 1,992 (92.9) | 1,035 (92.6) | 957 (93.2) |  |
| *Current ART regimen (N=2,100)* |  |  |  | <0.001 |
| 2A | 159 (7.6) | 69 (6.4) | 90 (8.8) |  |
| 5A | 1,746 (83.1) | 920 (85.7) | 826 (80.5) |  |
| 6A | 112 (5.3) | 38 (3.5) | 74 (7.2) |  |
| Other regimens* | 83 (9.0) | 47 (4.4) | 36 (3.5) |  |
| *ART duration (N=2,098)* |  |  |  | 0.242 |
| <24 months | 491 (23.4) | 262 (24.5) | 229 (22.3) |  |
| ≥ 24 months | 1,607 (76.6) | 809 (75.5) | 798 (77.7) |  |

*SD, standard deviation; IQR, interquartile range; ART, antiretroviral therapy; 2A = single tablet formulation of zidovudine, lamivudine, nevirapine; 5A = single tablet formulation of tenofovir, lamivudine, efavirenz; 6A = combined formulation of tenofovir and lamivudine plus single tablet of nevirapine*

** Others: 1A: single tablet formulation of stavudine, lamivudine, and nevirapine; 7A: combined formulation of tenofovir, lamivudine plus single tablet of atazanavir/ritonavir and 8A: combined formulation of zidovudine, lamivudine plus single tablet of atazanavir/ritonavir*
