# Supplementary material for: The burden of gynecomastia among men on antiretroviral therapy in Zomba, Malawi
Source: PLoS One. 2017 Nov 20;12(11):e0188379. doi: 10.1371/journal.pone.0188379 (PMC5695797; doi:10.1371/journal.pone.0188379)
Supplement: S2 Table — (DOCX) [file pone.0188379.s002.docx]

**Table 4:** Factors associated with gynecomastia among men on ART at Zomba Central Hospital

| **Characteristics** | **Gynecomastia** | | | | |
| --- | --- | --- | --- | --- | --- |
|  | **n (%) Gynecomastia** | **Crude OR**  **(95% CI)** | **P - value** | **Adjusted OR**  **(95% CI)*** | **P - value^¥^** |
| *Age in years (N = 1,027)* |  |  | 0.372 |  | 0.625  ^§^P^trend^  0.340 |
| 18 - 34 | 5 (3.9) | 1 |  | 1 |  |
| 35 - 44 | 22 (5.7) | 1.50 (0.56, 4.04) |  | 1.25 (0.46, 3.43) |  |
| ≥44 | 35 (6.9) | 1.85 (0.71, 4.82) |  | 1.51 (0.57, 4.03) |  |
|  |  |  |  |  |  |
| *BMI categories (N = 1,027)* |  |  | 0.479 |  |  |
| Underweight | 12 (7.7) | 1.43 (0.74, 2.79) |  |  |  |
| Normal | 43 (5.5) | 1 |  |  |  |
| Overweight | 7 (7.6) | 1.41 (0.61, 3.23) |  |  |  |
|  |  |  |  |  |  |
| *Baseline CD4 count at ART initiation (N=718)* |  |  | *0.064* |  | 0.170 |
| CD4 < 250 | 32 (7.2) | *1* |  | 1 |  |
| CD4 ≥250 | 9 (3.3) | *0.44 (0.21, 0.94)* |  | 0.51 (0.23, 1.12) |  |
| Missing | 21 (6.8) | *0.94 (0.53, 1.67)* |  | 1.02 (0.57, 1.81) |  |
|  |  |  |  |  |  |
| *ART duration in months (N=1,027)* |  |  | 0.112 |  |  |
| <24 | 9 (3.9) | 1 |  |  |  |
| ≥24 | 53 (6.6) | 1.74 (0.84, 3.58) |  |  |  |
|  |  |  |  |  |  |
| *WHO stage at ART initiation (N=812)* |  |  | 0.224 |  |  |
| Stage I/II | 23 (5.3) | 1 |  |  |  |
| Stage III/IV | 28 (7.4) | 1.42 (0.80, 2.51) |  |  |  |
|  |  |  |  |  |  |
| *History of TB treatment (N= 1,027)* |  |  | **0.022** |  | **0.040** |
| No | 51 (5.5) | **1** |  | **1** |  |
| Yes | 11 (12.1) | **2.39 (1.20, 4.76)** |  | **2.10 (1.04, 4.25)** |  |
|  |  |  |  |  |  |
| *Presence of lipodystrophy (N=1,027)* |  |  | 0.695 |  |  |
| No | 57 (6.0) | 1 |  |  |  |
| Yes | 5 (7.1) | 1.21 (0.47, 3.13) |  |  |  |
|  |  |  |  |  |  |
| *Current ART regimen (N = 1,026)* |  |  | **<0.001** |  |  |
| zidovudine, lamivudine, nevirapine (n=90) | 0 (0.0) | **-** |  |  |  |
| tenofovir, lamivudine, efavirenz (n=826) | 9 (1.1) | **1** |  |  |  |
| tenofovir, lamivudine, nevirapine (n=74) | 48 (64.9) | **167.59 (74.41, 377.47)** |  |  |  |
| Other regimens** (n=36) | 5 (13.9) | **14.64 (4.63, 46.27)** |  |  |  |
|  |  |  |  |  |  |
| *Exposure to efavirenz (N=1,026)* |  |  | 0.595 |  | 0.348 |
| No | 6 (5.0) | 1 |  | 1 |  |
| Yes | 56 (6.2) | 1.26 (0.53, 3.00) |  | 1.52 (0.63, 3.66) |  |
|  |  |  |  |  |  |
| *Exposure to stavudine (N=1,026)* |  |  | 0.128 |  | 0.576 |
| No | 16 (4.5) | 1 |  | 1 |  |
| Yes | 46 (6.9) | 1.57 (0.88, 2.82) |  | 1.19 (0.64, 2.22) |  |
|  |  |  |  |  |  |
| *Number of ART regimens (N=1,027)** |  |  | **<0.001** |  |  |
| One | 6 (1.8) | **1** |  |  |  |
| Two | 10 (1.8) | **0.98 (0.35, 2.73)** |  |  |  |
| Three | 38 (30.9) | **24.00 (9.82, 58.63)** |  |  |  |
| Four or more | 8 (40.0) | **35.78 (10.72, 119.40)** |  |  |  |

^¥^LHR test LRT test showing an overall p-value for age categories

§ p value for trend

*Final model included: age, baseline CD4 count, history of TB treatment, patient’s ART regimens include 1A and patient’s ART regimens include 5A

**Other regimens: stavudine, lamivudine, nevirapine; tenofovir, lamivudine and atazanavir/ritonavir; zidovudine, lamivudine and atazanavir/ritonavir
